# Electromyographic Comparison of Traditional and Suspension Push-Ups

**DOI:** 10.2478/hukin-2013-0070

**Published:** 2013-12-31

**Authors:** Ronald L. Snarr, Michael R. Esco

**Affiliations:** 1 School of Nutrition and Health Promotion, Arizona State University, Phoenix, AZ.; 2 Human Performance Laboratory, Department of Physical Education and Exercise Science, Auburn University at Montgomery, Montgomery, AL.

**Keywords:** EMG, instability devices, resistance training, TRX

## Abstract

There is very limited scientific data concerning suspension training. The purpose of this investigation was to compare the electromyographic activity of the pectoralis major, anterior deltoid, and triceps brachii between a suspension push-up and traditional push-up. Twenty-one apparently healthy men (n = 15, age = 25.93 ± 3.67 years) and women (n = 6, age = 23.5 ± 1.97 years) volunteered to participate in this study. All subjects performed four repetitions of a suspension push-up and a traditional push-up where the order of the exercises was randomized. The mean peak and normalized electromyography of the pectoralis major, anterior deltoid, and triceps brachii were compared across the two exercises. Suspension push-ups elicited the following electromyographic values: pectoralis major (3.08 ± 1.13 mV, 69.54 ± 27.6 %MVC), anterior deltoid (5.08 ± 1.55 mV, 81.13 ± 17.77 %MVC), and triceps brachii (5.11 ± 1.97 mV, 105.83 ± 18.54 %MVC). The electromyographic activities during the traditional push-up were as follows: pectoralis major (2.66 ± 1.05 mV, 63.62 ± 16.4 %MVC), anterior deltoid (4.01 ± 1.27 mV, 58.91 ± 20.3 %MVC), and triceps brachii (3.91 ± 1.36 mV, 74.32 ± 16.9 %MVC). The mean peak and normalized electromyographic values were significantly higher for all 3 muscles during the suspension push-up compared to the traditional push-up (p < 0.05). This study suggests that the suspension push-up elicited a greater activation of pectoralis major, anterior deltoid, and triceps brachii when compared to a traditional push-up. Therefore, suspension push-ups may be considered an advanced variation of a traditional push-up when a greater challenge is warranted.

## Introduction

The push-up (PU) is a popular exercise that is performed with the purpose of increasing strength and hypertrophy of upper extremity musculature (Dillman et al., 1994; Rogol et al., 1998; Ubinger et al., 1999; Uhl et al., 2003). It is also considered the standard measurement of upper-body muscular endurance (ACSM, 2008). Though the PU serves as an exercise to primarily target the pectoralis major (PM); it also activates the anterior deltoid (AD) and triceps brachii (TB) (Uhl et al., 2003; Youdas et al., 2010).

This exercise is traditionally performed on a flat, stable surface with hand placement at slightly wider than shoulder width. However, common variations exist involving changes in hand position from standard (e.g., wide or narrow) and modifying body posture by elevating the feet. A change in surface stability has recently been shown to also add variation and increased intensity of the PU. Most research in this area that has suggested that performing PU with instability devices such as Swiss balls, inflated discs, BOSUs and wobble boards may increase the activity of shoulder girdle and upper arm muscular compared to the traditional approach (Cogley et al., 2005; Contreras et al., 2012; Gouvali and Boudolos, 2005; Lehman et al., 2008; Youdas et al., 2010).

Suspension training (ST) is one of the newest forms of stability training that utilizes hanging ropes and straps that are anchored to a fixed point from above (e.g., ceiling or pull-up bar) allowing the user to work against their own body weight from a suspended position. Hypothetically, the greater disruption in stabilization from ST elicits increased motor unit recruitment, essentially causing the muscle to “work harder” to perform a particular movement (Beach et al., 2008; Marshall and Murphy, 2006). Unfortunately, limited scientific data exist regarding the effectiveness of this newer form of exercise. Two recent studies demonstrated that the PU performed on a suspension device elicited a greater activation of the rectus abdominis (Snarr et al., 2013) and latissimus dorsi (Beach et al., 2008) compared to a traditional stable PU. However, neither study examined the activity of the prime movers of the glenohumeral (e.g., PM and AD) and humeroulnar (e.g., TB) joints. Therefore, the purpose of this investigation was to determine the extent of electromyographic (EMG) activity of the PM, AD, and TB while performing push-ups with (SPUs) and without (PUs) a suspension device. As mentioned above, previous research has shown a greater EMG output of the selected muscles when performing the PU on common instability devices such as the Swiss ball (Cogley et al., 2005; Contreras et al., 2012; Gouvali and Boudolos, 2005; Lehman et al., 2008; Marshall and Murphy, 2006; Youdas et al., 2010). Therefore, it was hypothesized in the current study that SPUs would elicit a greater activation of the studied musculature compared to PUs.

## Material and Methods

There is increasing public interest on ST, yet limited scientific published data. Research is needed to determine the effectiveness of this newer form of exercise. This investigation was performed to compare the EMG activity of PM, AD, and TM between the SPU and PU. A group of subjects performed SPUs and PUs in randomized order. The EMG activity of the selected musculature was compared between the two trials. All measurements were taken on the same day. The complete details of the study are described in the following sections.

### Participants

Subjects were recruited through flyers and word of mouth. Subjects (n = 21) consisted of 15 men and 6 women who volunteered to participate in this study. Descriptive statistics for the participants are shown in Table 1. Participants were informed of all risks and discomforts that could occur and were asked to complete a health history questionnaire and informed consent. Only those who were classified as low risk, according to the American College of Sports Medicine guidelines were used in this study. Individuals with any previous chest, shoulder, or arm injuries were excluded from this investigation. All subjects were currently physically active with at least six months of resistance training experience. Concerning familiarity with ST, 18 subjects had no previous exposure, while 3 subjects were accustomed to regular exercise with an ST device. This study was approved by the Auburn University at Montgomery Institutional Review Board.

### Procedures: Electromyography

All EMG values were collected using a Biopac MP150 BioNomadix Wireless Physiology Monitoring system at a sampling rate of 1.000 kHz, and analyzed using Acqknowledge 4.2 software (BIOPAC System, Inc., Goleta, CA). Disposable Ag-AgCl surface electrodes (Biopac EL504) were used for this study. Before placing the surface electrodes, all skin sites were prepared with shaving, abasing, and alcohol cleansing in order to reduce impedance. All electrodes were placed on the right side of the subjects. Researchers assumed that bilateral symmetry was occurring throughout each exercise performed; therefore, electrodes were not placed on both sides of the subject. Pectoralis major electrodes were positioned halfway between the sternal notch and anterior axillary line, approximately 2 cm apart in-line with muscle fibers. Anterior deltoid electrodes were placed two finger-breadths below the acromio-clavicular joint and angled towards the deltoid tuberosity. The electrodes for the triceps brachii were positioned mid-way between the acromion and olecranon processes on the posterior portion of the upper arm on the long head of the tricep, approximately 2 cm apart following the muscle fibers. A ground electrode was placed directly over the right anterior-superior iliac spine. This method of electrode placement is similar to that of Cram and Kasman (1998).

### Exercise Trials

After all electrodes were placed, a maximum voluntary contraction (MVC) of each muscle group was determined to allow normalizations of the EMG data. To obtain normalization for the pectoralis major, subjects laid prone on a mat with elbows flexed to 90 degrees. A matched resistance was placed on the subjects’ upper back as they attempted to perform a push-up, resulting in a static maximal contraction. Next, the MVC for the triceps brachii was obtained by instructing the subject to assume a kneeling position with the upper arm resting on a bench and elbow flexed to 90 degrees. The subject then attempted to extend the elbow against a matched resistance. Lastly, the anterior deltoid EMG was normalized with the subject in a seated position with the shoulder flexed anteriorly to approximately 45 degrees. The subject then attempted to flex the shoulder against a matched resistance. This method of EMG normalization was performed in accordance with the standards set by Konrad (2005).

Once the EMG data was normalized, subjects drew numbers in order to randomize the exercises performed. All subjects were instructed on proper technique of the traditional and suspended push-up by a Certified Strength and Conditioning Specialist. If subjects were unable to complete the push-ups with proper technique, they were not used in the data collection process. The techniques for the exercises are as follows:

#### Suspension push-up (Picture 1)

Prior to performing the SPU, the suspension device was securely attached overhead to the top portion of a Smith Machine. In order to mimic the traditional PU, the handles of the suspension device were set to match the level of the feet when placed on a fitness step. The TRX® Suspension Trainer® was used for this investigation. Participants assumed a standard push-up position with hands placed in the handles of the suspension device (starting position). The hands were placed slightly wider than shoulder-width apart. Next, while maintaining a neutral spine and feet together position, subjects began the eccentric portion (descent) of the push-up. Suspension push-ups were only recorded when the correct depth was reached (chest reached the level of the hands) for each repetition. Push-ups were performed at a rate of 1 push-up every three seconds. Timing was measured by a metronome.

Eight jumps were removed prior to recalculating the correlation (data not shown).

#### Standard push-up (Picture 2)

Standard push-ups were performed on a flat, stable surface, hands placed slightly wider than shoulder-width apart, and fingers pointed forward. Subjects were instructed to maintain a neutral spine and feet together position throughout the entire movement. Once again, in order for the repetition to be recorded the correct depth needed to be met. Participants were instructed to lower the body until the chest was within 2 inches from the floor. All repetitions were repeated if the correct depth was not acquired. The same repetition timing was applied for all push-ups (1 push-up every 3 seconds).

### Statistical Analysis

Data was analyzed using SPSS/PASW Statistics version 18.0 (Somers, NY). Means and standard deviations were calculated for the studied variables (PM, AD, TB). Paired samples T-tests were used to determine if the mean peak (mV) and normalized (%MVC) EMG values for the PM, AD, and TB were significantly different between the PU and SPU. A priori statistical significance was set to a value of p < 0.05.

## Results

**Figure 1:**
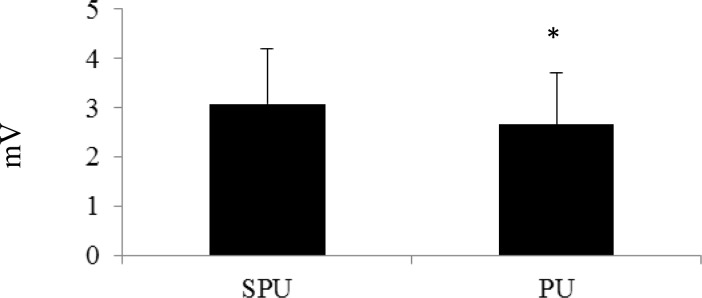
Comparison of Electromyographic Activity (mV) of the Pectoralis Major between Suspension Push-ups (SPU) and Traditional Push-ups (PU) *PU was significantly lower than SPU (p<0.05)

**Figure 2:**
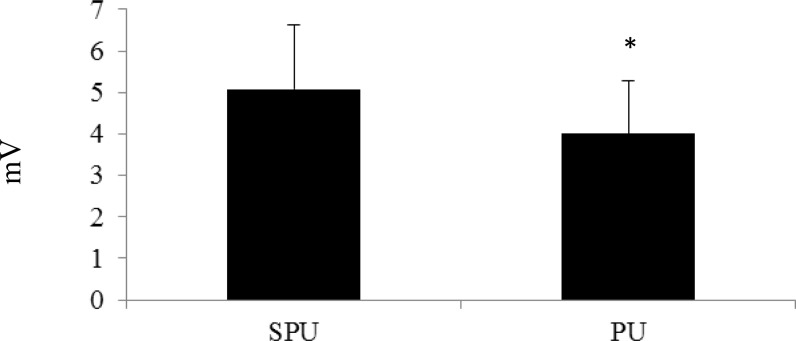
Comparison of Electromyographic Activity (mV) of the Anterior Deltoid between Suspension Push-ups (SPU) and Traditional Push-ups (PU) *PU was significantly lower than SPU (p<0.05)

**Figure 3:**
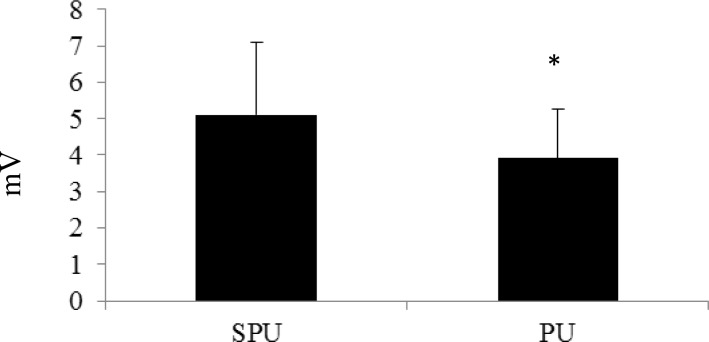
Comparison of Electromyographic Activity (mV) of the Triceps Brachii between Suspension Push-ups (SPU) and Traditional Push-ups (PU) *PU was significantly lower than SPU (p<0.05)

All of the subjects completed each exercise trial successfully and were included in the data collection process. The PM activity during the SPU and PU was 3.08 ± 1.13 mV and 2.66 ± 1.05 mV, respectively (Figure 1). The %MVC for the PM was 69.54 ± 27.6% during the SPU and 63.62 ± 16.4% during the PU. Activity for the AD during the SPU and PU was 5.08 ± 1.55 mV and 4.01 ± 1.27 mV, respectively (Figure 2). Normalized values for the AD were 81.13 ± 17.77% (SPU) and 58.91 ± 20.3% (PU). While, the TB activity for the SPU was 5.11 ± 1.97 mV and the PU was 3.91 ± 1.36 mV (Figure 3). The %MVC values during the SPU and PU were 105.83 ± 18.54% and 74.32 ± 16.9%, respectively. The EMG values (raw and normalized) for each muscle were all significantly higher during the SPU compared to the PU (p < 0.05).

## Discussion

The purpose of this study was to compare the EMG activity of the PM, AD, and TB between the SPU and PU. The major finding of this study was that the SPU resulted in significantly greater EMG activity (raw and normalized) of the selected muscles compared to the traditional PU. These results indicate that ST may be an effective method to increase the intensity of the standard PU when targeting the PM, AD, and TB.

The three muscles were chosen in this study because of their particular roles on glenohumeral and humeroulnar joint movement during the push-up. The PM is a uni-articulate muscle responsible for horizontal and diagonal adduction, along with internal rotation of the humerus. Various fibers of the PM (i.e., clavicular head) are also responsible for humeral flexion, while the sternocostal portion provides humeral extension (Floyd, 2009). While the entire deltoid provides-, multiple roles during the PU, the AD was chosen primarily for its role of humeral flexion, which is distinct to the anterior fibers (Floyd, 2009). The AD also provides horizontal and diagonal adduction, along with internal rotation of the humerus (Floyd, 2009). In addition, the TB is the primary concentric elbow extender during the PU (Floyd, 2009).

An abundance of research has examined the EMG activity of selected musculature while performing different exercises on various instability devices (Beach et al., 2008; Freeman et al., 2006; Marshall and Murphy, 2005; Marshall and Murphy, 2006; Marshall and Murphy, 2006). For example, the Swiss Ball has been shown to be an effective device for eliciting an increased level of muscular activity when used with exercises designed to target the PM, AD, and TB (Lehman et al., 2006; Marshall and Murphy, 2006; Marshall and Murphy, 2006). Our findings are consistent with previous research about the global topic of instability exercise; i.e., increased muscular activation during body weight exercise when stability is challenged (Anderson et al., 2011; Freeman et al., 2006; Lehman et al., 2006; Marshall and Murphy, 2005; Marshall and Murphy, 2006; Snarr et al., 2013). However, the current study is one of the first to suggest ST may be a superior method for increasing EMG activity of PM, AD, and TB. Several theories are available to help explain our findings, which are detailed within the following two paragraphs.

During a typical PU, each dynamically active joint has only one degree of freedom in which to function (i.e., a vertical, up-and-down movement). However, the ST decreases the base of support for the upper body, as it is suspended above the floor. This unstable kinetic chain results in additional degrees of freedom as the limbs work to prevent unnecessary horizontal and diagonal movements. This creates a “multiple-role” within the active musculature as they not only serve as PU agonists, but also as joint stabilizers (Lander et al., 1985; Marshall and Murphy, 2006; McCaw and Friday, 1994). The hands being placed in the handles of the suspension trainer provides additional degrees of freedom compared to the standard [fixed] floor placement. With additional ranges of freedom, a greater number of motor units is recruited to execute a particular exercise resulting in an increased EMG output (Beach et al., 2008; Behm, 1995; Marshall and Murphy, 2005; Marshall and Murphy, 2006; Vera-Garcia et al., 2000; Wahl and Behm, 2008). This characteristic is similar when performing dumbbell versus barbell chest presses, as the former has been shown to provide an increased level of instability (Behm, 1995; Saeterbakken et al., 2011). Furthermore, Saeterbakken et al. (2011) showed that with a shift from a one degree to a multiple degree of freedom bench press exercise (i.e., comparing barbells to dumbbells), EMG activation levels remained consistent in the primary musculature. However, the average load of the barbell bench press was 17% greater compared to the dumbbell bench press (Saeterbakken et al., 2011). In the current study, the participants performed both exercises while using the same load (i.e., their personal body weight) even though the degrees of freedom were greater with SPUs. Therefore, EMG output was higher.

In addition, previous research has shown that varying the position of the hands while performing a PU can lead to an increased EMG output of targeted musculature (Cogley et al., 2005; Youdas et al., 2010). Cogley et al. (2005) showed that when hands are placed narrower compared to wider than shoulder width, EMG output of the PM and TB is higher primarily due to a greater range of motion with the former. With the SPU, the hands are wider at the start and move to a more narrow position at the end of a concentric action. In contrast, the hands remained slightly wider than shoulder width throughout the PU movement. Therefore, the SPU resulted in a greater range of humeral motion compared to the PU, resulting in a greater EMG output of the selected glenohumeral musculature (i.e., PM and AD). Furthermore, narrow hand placement with PU has been shown to increase humeroulnar torque by 71% compared to a wider base (Donkers et al., 1993). Since the base of support is narrowed at the end of a concentric action with SPU, a greater EMG output of TB is also elicited, which is consistent with previous studies (Cogley et al., 2005; Donkers et al., 1993).

This study is not without possible limitations. First, the sample size had a diverse background with ST, with some subjects more familiar with this form of exercise compared to others. The EMG output of the selected muscular may decrease as familiarity with ST increases. A study performed by Wahl and Behm (2008) demonstrated that with highly resistance-trained individuals, not all instability devices were able to elicit significantly greater muscular activations during training. It may be warranted that future studies examine if EMG activation is different between individuals of various ST background levels. Second, only one device was used in this investigation (i.e., suspension device). A cross-comparison of multiple instability devices (e.g., swiss ball, wobble boards, etc.) may provide further insight into the overall effectiveness of ST. Third, a constant hand position (i.e., slightly wider that shoulder-width) was not maintained throughout a typical repetition of the SPU. Subjects began with a wider hand placement, but moved to a narrower placement at the end of the concentric action. This action is typical when performing a SPU due to the free-moving handles. Future study is warranted to determine the effect of various hand position widths on muscular activity during the SPU. Last, the group of subjects was not analyzed across a chronic training period. Longitudinal investigation is certainly needed before determining the effectiveness of ST on muscular hypertrophy and strength. However, the novel findings of the current study provide an important first step for future studies on ST.

Based on EMG values alone, our study indicates that the SPU exercise elicits greater muscular activation of PM, AD, and TB compared to the traditional PU. The traditional PU, when performed on a stable surface can provide a sufficient stimulus to increase upper body muscular strength and endurance (ACSM, 2008). However, when an increased challenge is warranted, a suspension training device may be incorporated to increase muscular activation and possibly enhance neuromuscular adaptations with the push-up. Therefore, practitioners should consider using ST for advancing the traditional push-up movement.

## Figures and Tables

**Picture 1:**
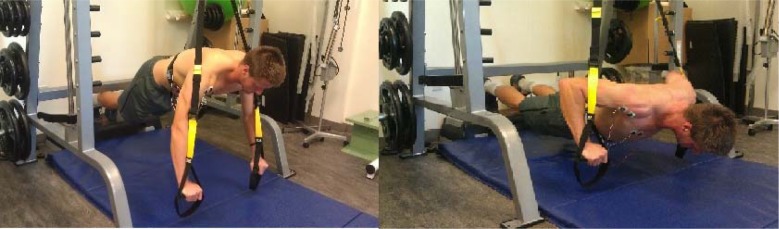
Starting and ending position for the suspension push-up (SPU)

**Picture 2:**
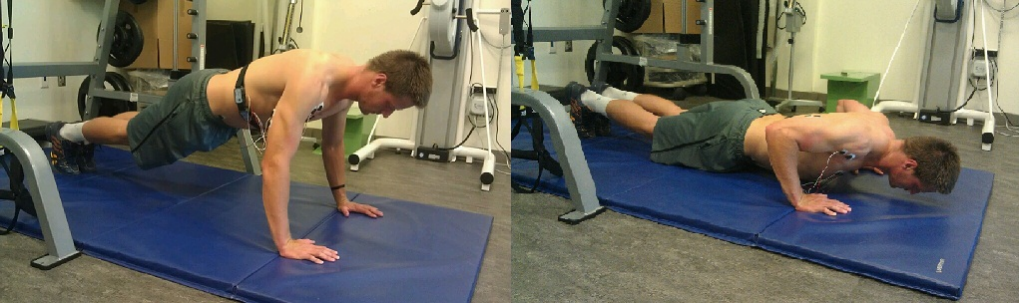
Starting and ending position for the traditional push-up (PU)

**Table 1:** Descriptive statistics of the study participants

	Men (n = 15)	Women (n = 6)	All (n = 21)
Age (yr)	25.93 ± 3.67	23.50 ± 1.97	25.24 ± 3.42
Height (cm)	180.78 ± 8.54	174.05 ± 4.96	179.01 ± 8.21
Body mass (kg)	83.65 ± 7.72	68.04 ± 6.56	79.54 ± 10.12
